# Twitter Trends for Celiac Disease and the Gluten-Free Diet: Cross-sectional Descriptive Analysis

**DOI:** 10.2196/37924

**Published:** 2022-12-05

**Authors:** Monique Germone, Casey D Wright, Royce Kimmons, Shayna Skelley Coburn

**Affiliations:** 1 Digestive Health Institute Colorado Center for Celiac Disease Children's Hospital Colorado Aurora, CO United States; 2 Department of Psychiatry University of Colorado Anschutz Medical Campus Aurora, CO United States; 3 Department of Developmental Sciences School of Dentistry Marquette University Milwaukee, WI United States; 4 David O McKay School of Education, Instructional Psychology & Technology Brigham Young University Provo, UT United States; 5 Department of Psychology and Behavioral Health Children’s National Hospital Washington, DC United States; 6 Department of Psychiatry and Behavioral Sciences The George Washington School of Medicine and Health Sciences Washington, DC United States

**Keywords:** celiac disease, social media, Twitter, gluten-free, social networking site, diet, infodemiology, education, online, content, accuracy, credibility

## Abstract

**Background:**

Few studies have systematically analyzed information regarding chronic medical conditions and available treatments on social media. Celiac disease (CD) is an exemplar of the need to investigate web-based educational sources. CD is an autoimmune condition wherein the ingestion of gluten causes intestinal damage and, if left untreated by a strict gluten-free diet (GFD), can result in significant nutritional deficiencies leading to cancer, bone disease, and death. Adherence to the GFD can be difficult owing to cost and negative stigma, including misinformation about what gluten is and who should avoid it. Given the significant impact that negative stigma and common misunderstandings have on the treatment of CD, this condition was chosen to systematically investigate the scope and nature of sources and information distributed through social media.

**Objective:**

To address concerns related to educational social media sources, this study explored trends on the social media platform Twitter about CD and the GFD to identify primary influencers and the type of information disseminated by these influencers.

**Methods:**

This cross-sectional study used data mining to collect tweets and users who used the hashtags #celiac and #glutenfree from an 8-month time frame. Tweets were then analyzed to describe who is disseminating information via this platform and the content, source, and frequency of such information.

**Results:**

More content was posted for #glutenfree (1501.8 tweets per day) than for #celiac (69 tweets per day). A substantial proportion of the content was produced by a small percentage of contributors (ie, “Superuser”), who could be categorized as self-promotors (eg, bloggers, writers, authors; 13.9% of #glutenfree tweets and 22.7% of #celiac tweets), self-identified female family members (eg, mother; 4.3% of #glutenfree tweets and 8% of #celiac tweets), or commercial entities (eg, restaurants and bakeries). On the other hand, relatively few self-identified scientific, nonprofit, and medical provider users made substantial contributions on Twitter related to the GFD or CD (1% of #glutenfree tweets and 3.1% of #celiac tweets, respectively).

**Conclusions:**

Most material on Twitter was provided by self-promoters, commercial entities, or self-identified female family members, which may not have been supported by current medical and scientific practices. Researchers and medical providers could potentially benefit from contributing more to this space to enhance the web-based resources for patients and families.

## Introduction

Chronic disease diagnoses often are coupled with a significant period of adjustment as patients learn how to manage and live with the condition. Having access to relevant and reliable information is important for educating and aiding new patients in disease management [1-3]. Over the past 16 years, many individuals with a chronic disease have been turning to Internet sources, such as social media, for education about their condition and treatment [4,5] despite a hesitancy from physicians and medical providers to use this resource for patient education [6]. Social media use among Americans has increased dramatically across adults of all genders, race, income, education level, and communities since the early 2000s [4]. The social media platform Twitter provides a unique data source whereby important questions can be asked and analyzed regarding how various participants are searching and sharing information, such as information related to patient education and disease management.

Having the technological ability to collect (ie, “mine”) publicly available data on social media platforms such as Twitter provides an opportunity to systematically quantify and categorize information on such platforms into trends and useful information for interested parties (eg, patients with chronic diseases). One component of using these emerging methodologies to analyze social media information is through the use of “affinity spaces.” Affinity spaces represent either physical or web-based gathering places (rather than geographic or identity-based communities) where people come together in a “common endeavor” to develop and share various types of knowledge, including individual, internal, and in-depth information [7].

The systematic application of common data mining techniques on social media platforms facilitates the analysis of disease management–related trends and information available to patients [5]. This is of relevance to those with celiac disease (or in British English, “coeliac disease”; CD). CD is a condition that requires extensive education around a dietary treatment steeped in stigma and myth [8]. CD is a chronic autoimmune condition wherein the ingestion of gluten results in an immune-mediated injury to the small intestine [9]. Damage to the small intestine leads to malabsorption of nutrients and can result in short- and long-term complications ranging from gastroenterological distress to cancer and even death [9]. It is estimated that CD affects approximately 1% of individuals worldwide [10]. To date, the only treatment is adherence to a strict gluten-free diet (GFD) [11]. CD is associated with heavy biopsychosocial demands and challenges following a CD diagnosis [12,13].

Prior work on broader internet-based sources for CD education is emerging and denotes concern for the information, and misinformation, that is presented by these sources [14-18]. Overall, information disseminated by the top websites found in web-based searches conducted by researchers are not entirely accurate, transparent, or reliable for interested consumers such as patients or providers, including dietitians [15,17,19]. Moreover, despite its potential to reach millions of viewers, the top videos on YouTube related to CD in 2019 lacked adequate or helpful information [14].

Given the high prevalence of CD and the heavy burden associated with managing CD and the GFD, many resources are available; nonetheless, it is difficult to identify credible educational information about the treatment for CD (a GFD). New methodologies from the field of computer science have emerged that allow for further exploration of patient education through not only the internet but also, more specifically, the social media space. The purpose of this study was to combine the fields of computer science and behavioral science to explore trends on Twitter as an educational source for patients with CD. This study conducted a preliminary evaluation of the scope and nature of information available on Twitter by (1) determining who the primary contributors are who lead the conversations about CD and GFD-related topics on Twitter, as well as (2) identifying what type of information (ie, content, source, and frequency) is being disseminated by these contributors.

## Methods

### Selecting an Internet Information Source

The social media platform Twitter allows for broader access to data than other social media platforms. Additionally, the nature of “tweets” (posts from Twitter users) and user profile descriptors is text-based versus image-based (such as content found on Instagram), which allows for more ready analysis of the data. Despite not being the most widely used platform, as is YouTube (81%) or Facebook (69%), Twitter is used by approximately a quarter (23%) of American adults and relatively equally among self-identified men and women and racial groups [5]. A 2021 survey of US adults demonstrated that young adults (18 to 29 years) are the predominant users of social media [5]. However, use by older adults (>65 years) has increased in recent years to 45% of older adults in 2021, which indicates that they use at least 1 social media site [5]. Given the ready availability of the data and wide use of users including individuals with CD, Twitter was chosen as the social media source for this study.

### Defining Affinity Spaces

An increasingly common research practice has been to examine affinity spaces found on the popular social media platform Twitter through the use of hashtags (an author’s use of the hash symbol followed by the subject of a message) as a way to categorize and group messages; eg, #celiac and #glutenfree) [20,21]. These hashtags are conceptualized as a type of affinity space to explicate how these organic web-based spaces are used by communities to communicate, share, and find information [20,21]. As an open platform with very few barriers to participation and 330 million monthly active users [22], Twitter encourages the organic development of affinity spaces around topics and events via hashtagged keywords (eg, #celiac and #glutenfree).

The 2 topics most central to this study are “celiac” and “gluten-free.” Information available on Twitter regarding these topics might exhibit different norms in terms of who participates in these affinity spaces and how (eg, someone might want information on a gluten-free diet for non–celiac-related reasons). Hence, the original tweets that were tagged by Twitter users with either the #celiac or #glutenfree hashtag were treated as 2 different affinity spaces rather than 1 collective affinity space. These affinity spaces were then analyzed individually and compared to each other. Moreover, recognizing that many other hashtags might be used synonymously with #glutenfree or #celiac, hashtags akin to either of these terms in their relative affinity spaces also were included (ie, #gluten-free, #glutenfreediet, and #gluten_free, with #glutenfree and including #celiacdisease, #celiacs, #celiacsdisease, #coeliac, and #coeliacdisease with #celiac).

### Data Collection

This study collected Twitter user and tweet data using the public Twitter application programming interface. Researchers used custom PHP scripts to collect query results and store them to a MySQL relational database for cleaning and analysis. The data set used in this study consisted of tweets that included any of the hashtags listed above. Given the large number of such tweets, we limited the time frame of our study to 8 months (October 27, 2019, through June 8, 2020), allowing us to have sufficient data for analysis without being influenced too heavily by a single event (eg, the US Thanksgiving holiday season or the onset of the COVID-19 pandemic). Furthermore, because our main emphasis was to understand who was posting to these hashtags, “retweets” (a user who reposts a message created by a different user) were excluded to focus only on original posts and the users who generated them. This resulted in 334,907 and 15,602 original tweets containing #glutenfree and #celiac, respectively, including those from synonymous hashtags for analysis. Table 1 provides an overview of general user and Tweet metadata over the 8-month collection period.

**Table 1:** General user and tweet metadata over the 8-month data collection period.

	#glutenfree^a^				#celiac^b^		
Metric	Superuser	Contributor	Lurker	Superuser		Contributor	Lurker
User count, n	1718	16,947	145,246	44		394	3945
Overall tweets, %	25.5	25.2	49.3	28.7		35.2	36.1
Tweets per user, mean (SD)	49.8 (84.0)	5.0 (2.7)	1.1 (0.3)	101.7 (58.7)		13.9 (9.9)	1.4 (0.9)

^a^Tweets: n=334,907; tweets per user: mean 2.0, SD 10.0; users: n=163,911.

^b^Tweets: n=15,602; tweets per user: mean 3.6, SD 3.6; users: n=4383.

### Data Analysis

As is standard in analyzing data gathered from Twitter to analyze affinity spaces [20,21], all tweet and author users’ publicly available profile data (eg, Twitter handles and locations) were saved to a database. Descriptive statistics of tweet and author user objects were calculated to determine the method to use to classify users into user types for further analysis. Descriptive statistics revealed that users exhibited a highly positive skew in their posting activities. This behavior was expected given previous studies carried out on Twitter data [23]. Based on the positive skew, van Mierlo’s [24] 90-9-1 Principle was selected to classify users in each affinity space into relative activity groups. Users were classified as follows: superusers (top 1% of users posting content), contributors (next 9% of users contributing content), or lurkers (the remaining 90% of users; see Table 1) [24]. Following the standard for affinity space analysis [20,21], basic language processing techniques were then used to (1) extract keywords from user biographies (eg, “doctor” or “blogger”), (2) identify co-occurring hashtags (eg, “#vegan” or “#recipe”), and (3) identify common domains that users linked to in their tweets (eg, celiac.com). A detailed description of these categories is provided below in the *Results* section.

### Ethical Considerations

Ethics approval was obtained or determined to not be necessary by all author institutions owing to the public nature of the data.

## Results

### Aim 1: Examining the Primary Influencers on Twitter

#### User Activity Group: Superusers, Contributors, and Lurkers

Participation in each affinity space (ie, #glutenfree and #celiac) was evenly spread across the 3 groups, with superusers producing 25.5% of an overall 28.7% of posts containing #glutenfree and #celiac, contributors producing 25.2% of an overall 35.2% of posts, and lurkers producing 49.3% of an overall 36.1% posts. In other words, superusers (1% of users posting to the named affinity spaces) posted on average 10.0 times (#glutenfree) and 7.3 times (#celiac) more than contributors (the next 9% of users contributing), and contributors posted 4.5 times (#glutenfree) and 9.8 times (#celiac) more than lurkers (the other 90% of users posting to these spaces). Additionally, a comparison of raw tweet counts showed that Lurker behaviors were similar between the 2 hashtag groups but that #celiac superusers and contributors posted at least twice as often as their #glutenfree counterparts. #glutenfree represented more than 20 times the tweets as #celiac, but 40.3% of tweets in #celiac were also cross-listed in the #glutenfree data set (Table 1).

#### Biographical Self-descriptors

To understand the professional backgrounds of Twitter users posting to these hashtags, each user’s self-description was parsed out into a list of keywords [25] after removing stop words (eg, “a,” “and,” and “the”). Descriptions produced roughly 200,000 unique keywords (eg, “blogger” and “author”). The study team reviewed the most common 500 keywords for each hashtag and user activity group and then excluded those that did not suggest the author’s expertise or were disassociated from the topic (eg, “director” and “vegan” were retained, while “music” and “www” were excluded). Descriptors related to family relationships were also retained (eg, “mother” was included), expecting that many family members of individuals with CD would participate in these affinity spaces to learn more about managing CD and the GFD. Specifically, tweets from users who self-identified with these keywords related to female family relationships (eg, mother or wife) represented 4.3% of tweets containing #glutenfree and 8% of those containing #celiac. Male family relationships (eg, father or husband) represented 1.5% of tweets containing #glutenfree and 1.2% of those containing #celiac.

Specific keywords that suggested an author’s medical expertise (eg, “doctor,” “physician,” or “dietitian”) or a terminal degree (eg, “MD” and “PhD”) were also targeted [25]. Top results for each hashtag (#glutenfree and #celiac) and user category are provided in Tables 2 and 3; they indicated that “writer” (3.6% and 4.5%), “blogger” (1.4% and 2.4%), “author” (1.8% and 3.2%), and “advocate” (0.8% and 2.3%) were some of the most common self-descriptors. Targeted medical degrees and the term “doctor” were not widely used as self-descriptors by users and are provided in Tables 4 and 5, with “writers” and “bloggers” typically out-representing “PhDs” and “MDs” at a rate of 10-to-1 or more. The word stems “naturopath-” and “homeopath-” also accompanied many instances of “doctor” in both hashtags (5.5% and 14.0%, respectively). Overall, tweets from users who self-identified with keywords including “doctor,” “dietitian,” “physician,” “PhD,” or “MD” represented only 2.0% of tweets containing #glutenfree and 6.0% of those containing #celiac.

Recognizing that some users might identify terminal degrees and medical expertise in their name fields instead of their descriptions, a keyword search for variants of “Doctor,” “Physician,” “PhD,” “MD,” and “dietitian” on names was conducted. This showed that 0.4% of #glutenfree users and 2.1% of #celiac users self-identified with one of these terms in this way, but this calculation also included various distractors, such as multiple references to the television series “Doctor Who.”

**Table 2:** Top 15 self-descriptive identifiers of user accounts posting to #glutenfree.

	Superuser (n=1718)				Contributor (n=16,947)					Lurker (n=145,246)		
Rank	Keyword	Posts, n			Keyword		Posts, n		Keyword		Posts, n	
1	Blogger		109	Writer		718		Writer				4432
2	Vegan		103	Vegan		626		Lover				3883
3	Writer		65	Lover		543		Fan				3646
4	Author		64	Mom		511		Mom				3069
5	Mom		56	Blogger		458		Artist				2392
6	Lover		53	Author		434		Wife				2309
7	Creator		36	Fan		379		Author				2235
8	Chef		34	Artist		372		Enthusiast				2060
9	Foodie		32	Wife		365		Vegan				1744
10	Photographer		27	Enthusiast		238		Husband				1664
11	Fan		26	Chef		237		Student				1589
12	Wife		25	Mother		196		Blogger				1531
13	Owner		23	Photographer		188		Teacher				1465
14	Advocate		22	Owner		178		Father				1376
15	Coach		21	Coach		177		Mother				1358

**Table 3:** Top 15 self-descriptive identifiers of users posting to #celiac.

	Superuser (n=44)			Contributor (n=394)			Lurker (n=3945)		
Rank	Keyword	Posts, n	Keyword		Posts, n	Keyword		Posts, n	
1	Blogger	5	Mom		27	Mom			156
2	Advocate	4	Blogger		23	Lover			150
3	Vegan	4	Advocate		21	Writer			149
4	Author	3	Writer		20	Wife			123
5	Mom	2	Wife		17	Author			101
6	Writer	2	Lover		17	Fan			95
7	Wife	2	Vegan		16	Mum			78
8	Mother	2	Author		16	Advocate			75
9	Chef	2	Dietitian		10	Blogger			69
10	Host	2	Mother		8	Husband			68
11	Dietitian	1	Editor		8	Vegan			67
12	Editor	1	Founder		8	Student			63
13	Mum	1	Physician		7	Mother			61
14	MD	1	Fan		6	Teacher			58
15	Teacher	1	Student		6	Dietitian			52

**Table 4:** Targeted medical degrees or terms that are self-descriptive identifiers of user accounts posting to #glutenfree.

Superuser (n=1718)		Contributor (n=16,947)					Lurker (n=145,246)		
Keyword	Posts, n	Keyword		Posts, n	Keyword			Posts, n	
Dietitian	11	PhD	80			PhD			762
PhD	4	Dietitian	55			Doctor			347
MD	2	Doctor	45			Dietitian			187
Doctor	2	MD	22			MD			169
Physician	0	Physician	16			Physician			97

**Table 5:** Targeted medical degrees or terms that are self-descriptive identifiers of user accounts posting to #celiac.

Superuser (n=44)		Contributor (n=394)		Lurker (n=3945)	
Keyword	Posts, n	Keyword	Posts, n	Keyword	Posts, n
Dietitian	1	Dietitian	10	Dietitian	59
MD	1	Physician	7	PhD	46
Doctor	0	PhD	4	Doctor	29
PhD	0	MD	3	MD	14
Physician	0	Doctor	2	Physician	14

### Aim 2: Examining the Type of Information Distributed on Twitter

#### Affinity Spaces: #glutenfree Versus #celiac

Comparing the 2 affinity spaces, #glutenfree was much more active, averaging 1501.8 (SD 223.2) tweets per day, while #celiac averaged 69.0 (SD 16.7) tweets per day. Users posting to #glutenfree represented 163,911 accounts, averaging 2.0 (SD 10.0) tweets per account for the time period, while users posting to #celiac represented 4383 accounts, averaging 3.6 (SD 12.4) tweets per account. At the user participation level, a noticeable overlap was found between affinity spaces, with 64.0% of #celiac posters also posting to #glutenfree in the time period (with 1.7% of #glutenfree users also posting to #celiac).

#### Co-occurring Hashtags

To better understand the nature of the tweets that were being posted in each affinity space, the use of co-occurring hashtags was analyzed for easy grouping. In other words, hashtags that were used in tweets that did not have similar word stems to the targeted grouping hashtags (eg, #vegan was included in #glutenfree, while #gluten and #gf were ignored) were analyzed to identify groupings [26]. Percentages for each co-occurring hashtag were calculated by the likelihood that the hashtag would be used if any co-occurring hashtags existed at all (see Tables 6 and 7).

Tweets containing #celiac were highly represented in the #glutenfree data set, ranking at a similar level to mentions of paleo and keto diet hashtags, but overall results indicate that tweets containing #glutenfree focused heavily on a variety of other diets, including #vegan, #dairyfree, #plantbased, #keto, #paleo, #vegetarian, and #organic, suggesting that interest in GFDs was most commonly associated with a variety of weight loss and health regimens unrelated to CD (Tables 6 and 7). In the #celiac data set, gluten-related hashtags were dominant (with #glutenfree co-occurring in 50.5%-69% of tweets; see Tables 6 and 7), but other hashtags were more varied with some focusing on recipes (eg, #veganrecipes), others on symptoms (eg, #chronicpain), and other diseases (eg, #IBD and #IBS). These hashtags amounted to less than 1% of overall tweets.

Comparing the 2 affinity spaces, it appeared that #glutenfree was both more widely used but also more lifestyle based (eg, associated with other diet trends such as paleo or keto) than the #celiac space (see Tables 6 and 7).

**Table 6:** Top 15 co-occurring hashtags with #glutenfree.

	Superuser		Contributor		Lurker	
Rank	Hashtag	Posts, %	Hashtag	Posts, %	Hashtag	Posts, %
1	Vegan	20.9	Vegan	18.5	Vegan	13.2
2	Recipe	7.5	Dairyfree	6.4	Dairyfree	3.6
3	Dairyfree	7.4	Keto	2.6	Keto	1.8
4	Recipes	5.6	Celiac	2.5	Food	1.7
5	Food	5.3	Plantbased	2.5	Organic	1.7
6	Cooking	4.5	Recipe	2.3	Vegetarian	1.5
7	Keto	4.3	Paleo	2.3	Celiac	1.5
8	Lowcarb	4.0	Organic	2.2	Plantbased	1.5
9	Paleo	4.0	Vegetarian	2.1	Sugarfree	1.4
10	Celiac	3.5	Food	1.9	Baking	1.4
11	Delicious	3.3	Healthy	1.7	Healthy	1.3
12	Vegetarian	3.0	Lowcarb	1.6	Paleo	1.2
13	Cook	2.9	Coeliac	1.5	Recipe	1.1
14	Organic	2.6	Homemade	1.5	Homemade	1.0
15	Foodie	2.3	Sugarfree	1.5	Pizza	1.0

**Table 7:** Top 15 co-occurring hashtags with #celiac.

	Superuser			Contributor			Lurker	
Rank	Hashtag	Posts, %	Hashtag		Posts, %	Hashtag		Posts, %
1	GlutenFree	69.0	GlutenFree		66.3	GlutenFree		50.5
2	Gluten	14.5	Gluten		7.5	Gluten		9.0
3	Foodpics	12.7	Vegan		4.1	Vegan		4.4
4	Lovefood	12.3	Food		3.1	GF		2.5
5	Foodies	11.9	Foodie		3.0	Autoimmune		2.5
6	Vegan	10.4	GF		2.8	IBS		1.7
7	Freefrom	6.9	GlutenFreeLife		2.6	Covid19		1.7
8	Veganfood	6.9	Autoimmune		2.5	Disease		1.5
9	Health	6.8	Covid19		2.1	Dairyfree		1.5
10	Veganfriendly	6.8	Singluten		2.0	Health		1.5
11	Eggallergy	6.8	Dairyfree		1.9	Coronavirus		1.5
12	Veganrecipes	6.5	Colesbakeryandcafe		1.9	Foodallergy		1.4
13	Veganfoodlover	6.4	Freefrom		1.8	IBD		1.3
14	Eggfreefood	6.4	Foodallergies		1.8	Food		1.3
15	Chronicpain	6.3	Beer		1.7	Foodallergies		1.3

#### Shared Link Domains

To understand what resources users were sharing, the domains of unshortened links in tweets were analyzed. URL shorteners that were used as aliases rather than an actual direct link, and automated content providers were ignored (eg, bit.ly) [27]. Results for both affinity spaces revealed that links to social media and video sharing sites were common (eg, Instagram, Pinterest, and YouTube), and many blog, recipe, and other specialty sites were heavily linked to as well (see Table 8). Some of these domains were highly represented because many users were tweeting about them (eg, 1064 users tweeting YouTube videos in posts containing #glutenfree), but others were highly represented because a relatively small number of users were promoting a specific resource (eg, 1 user tweeting about foodgawker.com 136 times and promoting it to the #2 spot; Table 8).

Domains ending in “.com” (ie, commercial sites) were more prevalent (as opposed to nonprofit [.org] or government [.gov] domains). In fact, keyword searches for .com, .org, and .gov domains on the overall data set revealed that .com websites were linked to posts containing #glutenfree or #celiac 54.7 and 16.8 times more than .org sites and 1173.0 and 44.7 times more than .gov domains. This shows that the commercial influence seems to be much more apparent and disproportional to other influences in the #glutenfree space but that information in the #celiac space may also be heavily dominated by commercial interests.

**Table 8:** Most common linked domains.

#glutenfree				#celiac		
Domain	Tweets, n	Unique users, n	Domain		Tweets, n	Unique users, n
instagram.com	4446	2385	celiac.com		172	9
pinterest.com	2245	91	foodgawker.com		136	1
youtu.be	1924	1064	instagram.com		98	59
celiac.com	1454	18	wp.me		44	1
goo.gl	812	101	paper.li		34	10
simplygluten-free.com	632	42	youtu.be		30	24
untp.beer	534	474	gofundme.com		28	3
recipecialist.com	532	1	mygfguide.com		26	1
bloglovin.com	521	137	joshealthykitchen.com		22	1
amzn.to	475	118	theglutenfreeblogger.com		22	1
ntelikanis.com	465	1	glutenfreerespect.com		21	3
wp.me	437	24	ncbi.nlm.nih.gov		21	4
thisvivaciouslife.com	375	47	facebook.com		16	12
amazon.com	326	109	hamandeggerfiles.blogspot.com		16	2
sumo.ly	306	57	coeliac.org.uk		15	8
youtube.com	303	164	parenting.nytimes.com		12	11
facebook.com	255	192	drrobertpastore.com		12	1
mummytries.com	217	1	michellesglutenfreekitchen. wordpress.com		11	1
lifewaysvillage.com	206	3	medicalxpress.com		10	6
growingupgf.com	187	1	glutenfreepan.com		10	1

## Discussion

### Principal Findings

The purpose of this study was to combine methods from computer science and the behavioral sciences to begin to examine internet-based CD educational sources. As part of this initial investigation, this study describes information about CD and the GFD disseminated on the social media platform Twitter. With increasing use of social media as an educational resource and source of support for populations of individuals with chronic illness [28-30], it is crucial to understand the nature of information on platforms such as Twitter. Our findings emphasize the prominence of posts on both CD and the GFD, which appear to come from users focused on promotion of themselves (eg, identifying as vegan) or a business (eg, endorsing a restaurant) rather than from more traditional sources of information such as medical professionals or nonprofit organizations [19]. This supports previous findings regarding the hesitancy of medical providers to engage in social media as a form of medical education [6]. It also raises concerns about the quality of information individuals are receiving about CD and the GFD, as individuals with CD require the GFD for medical purposes [19]. This is likely not unique to CD as concerns have been raised in the field of food allergies [30]. We propose the need for a social media presence focused on providing high-quality, up-to-date, fact-checked information to users, particularly for those within the CD or other gluten-related diseases.

### Clinical Implications

Based on our findings, there is an opportunity and arguably a demand for increased presence on social media and internet-based platforms among medical and nonprofit experts in CD to provide high-quality information to consumers. This has been executed among populations of individuals with other diseases, such as inflammatory bowel disease (IBD). For example, ImproveCareNow [31] is a community of clinicians, researchers, parents, and patients of children and youths with IBD. The main goal of this organization is to provide a platform to help this community learn about “more reliable, proactive IBD care” [31]. Their social media campaign involves accounts on various platforms, including a blog, Facebook, Twitter, and YouTube. The content posted on these platforms is monitored by the organization.

Guidelines have been developed by several organizations to help inform medical providers on social media best practices, including the Association for Healthcare Social Media [32]. The use of guidelines can best inform medical providers on the use of social media as a source of patient education. Other groups are working to develop competencies including advocacy and communication responsibilities that specialists in various areas of health might develop in helping to educate certain patient populations [33].

### Limitations and Future Directions

There are several additional considerations for this study in analyzing publicly available Twitter data. First, we collected our sample of data during a relatively narrow (8-month) time period, which may not account for natural variations across seasons and events (eg, holidays and major scientific or medical conferences). The activity and nature of posts may have changed as the COVID-19 pandemic has continued. Second, our analysis did not examine co-occurring words within individual user accounts. For instance, it is possible that one account may note being a “vegan,” “blogger,” and “mom.” Future research could collect more detailed information about active members of social media to better understand “influencers” in this area. Furthermore, this study should be understood in light of the typical Twitter user. Twitter is used by about a quarter of American adults, both men and women of various racial groups, but we recognize that social media users may be younger and not necessarily representative of all ages and demographics [5]. Future work might examine the role of social media use in educating different subgroups of the population.

Additionally, we used established but relatively new methods of automated extraction and categorization of data rather than human coding, though we used human observation and judgment during the process of cleaning and synthesizing the data. This relied on algorithms based on anticipated data and did not allow for inductive reasoning by the human eye. Such an approach allowed the study team to rely on objective data rather than potential biases or a priori assumptions of individual experts [34]. Future studies may strengthen knowledge on this topic through expansion of data collection across a longer time span and further evaluation of the nature of users as well as the sentiments and accuracy of content within tweets.

### Conclusions

To our knowledge, this was the first study evaluating Twitter data using the topics #celiac and #glutenfree. Given the popularity and broad use of social media, this is an important starting point for this research that generates several new hypotheses and research questions. Our findings emphasize the large volume of information communicated on social media. We suggest that platforms such as Twitter pose risks of spreading biased or inaccurate information to the public, particularly when the sources of information come from entities who may be influenced by commercial conflicts of interest.

Social media represents an immense opportunity to achieve open and clear dialogue between health care professionals and the public, which could be a major facilitator of future research and patient education about CD and the GFD.

## Abbreviations

CD: celiac disease
GFD: gluten-free diet
IBD: inflammatory bowel disease

## References

[1] Stenberg U, Haaland-Øverby M, Koricho AT, Trollvik A, Kristoffersen LR, Dybvig S, Vågan A (2019). How can we support children, adolescents and young adults in managing chronic health challenges? A scoping review on the effects of patient education interventions. Health Expect.

[2] Pinchera B, DelloIacono D, Lawless CA (2018). Best practices for patient self-management: implications for nurse educators, patient educators, and program developers. J Contin Educ Nurs.

[3] Win KT, Hassan NM, Oinas-Kukkonen H, Probst Y (2016). Online patient education for chronic disease management: consumer perspectives. J Med Syst.

[4] (2021). Social Media Fact Sheet. Pew Research Center.

[5] Chirumamilla S, Gulati M (2021). Patient education and engagement through social media. Curr Cardiol Rev.

[6] Katz M, Nandi N (2021). Social media and medical education in the context of the COVID-19 pandemic: scoping review. JMIR Med Educ.

[7] Gee J, Barton D, Tusting K (2005). Semiotic social spaces and affinity spaces: From The Age of Mythology to today's schools. Beyond Communities of Practice: Language Power and Social Context (Learning in Doing: Social, Cognitive and Computational Perspectives).

[8] Pytell R (2017). More than a diet: the struggles of celiac disease. Dialouges@RU.

[9] Rubin JE, Crowe SE (2020). Celiac disease. Ann Intern Med.

[10] Singh P, Arora A, Strand TA, Leffler DA, Catassi C, Green PH, Kelly CP, Ahuja V, Makharia GK (2018). Global prevalence of celiac disease: systematic review and meta-analysis. Clin Gastroenterol Hepatol.

[11] Treatment for celiac disease. How do doctors treat celiac disease?. National Institute of Diabetes and Digestive and Kidney Diseases.

[12] Berry N, Vaiphei K, Dhaka N, Sinha SK, Kochhar R (2018). Quality of life in celiac disease and the effect of gluten-free diet. JGH Open.

[13] Theethira TG, Dennis M (2015). Celiac disease and the gluten-free diet: consequences and recommendations for improvement. Dig Dis.

[14] Basch CH, Hillyer GC, Garcia P, Basch CE (2019). Content of widely viewed YouTube videos about celiac disease. Public Health.

[15] England CY, Nicholls AM (2004). Advice available on the Internet for people with coeliac disease: an evaluation of the quality of websites. J Hum Nutr Diet.

[16] McNally SL, Donohue MC, Newton KP, Ogletree SP, Conner KK, Ingegneri SE, Kagnoff MF (2012). Can consumers trust web-based information about celiac disease? Accuracy, comprehensiveness, transparency, and readability of information on the internet. Interact J Med Res.

[17] Tangri V, Chande N (2011). Quality of Internet-based information on gastrointestinal diseases. Can J Gastroenterol.

[18] van der Marel S, Duijvestein M, Hardwick JC, van den Brink GR, Veenendaal R, Hommes DW, Fidder HH (2009). Quality of web-based information on inflammatory bowel diseases. Inflamm Bowel Dis.

[19] Buseck A, Lebwohl B, Green PHR (2021). Quality and content of online patient resources for celiac disease. Dig Dis Sci.

[20] Greenhalgh SP, Koehler MJ (2016). 28 Days later: Twitter hashtags as “Just in Time” teacher professional development. TechTrends.

[21] Rosenberg JM, Greenhalgh SP, Koehler MJ, Hamilton ER, Akcaoglu M (2016). An investigation of State Educational Twitter Hashtags (SETHs) as affinity spaces. E-Learning and Digital Media.

[22] Lin Y (2022). 10 Twitter statistics every marketer should know in 2022. Oberlo.

[23] Veletsianos G, Kimmons R (2016). Scholars in an increasingly open and digital world: how do education professors and students use Twitter?. Internet High Educ.

[24] van Mierlo T (2014). The 1% rule in four digital health social networks: an observational study. J Med Internet Res.

[25] Carpenter JP, Kimmons R, Short CR, Clements K, Staples ME (2019). Teacher identity and crossing the professional-personal divide on twitter. Teach Teach Educ.

[26] Weng L, Menczer F (2015). Topicality and impact in social media: diverse messages, focused messengers. PLoS One.

[27] Kimmons R, Veletsianos G, Woodward S (2016). Institutional uses of Twitter in U.S. higher education. Innov High Educ.

[28] Hamshaw RJ, Barnett J, Lucas JS (2017). Framing the debate and taking positions on food allergen legislation: the 100 chefs incident on social media. Health Risk Soc.

[29] Hamshaw RJT, Barnett J, Lucas JS (2018). Tweeting and eating: the effect of links and likes on food-hypersensitive consumers' perceptions of Tweets. Front Public Health.

[30] Hamshaw RJT, Barnett J, Gavin J, Lucas JS (2019). Perceptions of food hypersensitivity expertise on social media: qualitative study. Interact J Med Res.

[31] ImproveCareNow.

[32] Association for Healthcare Social Media.

[33] Stellefson M, Paige SR, Chaney BH, Chaney JD (2020). Evolving role of social media in health promotion: updated responsibilities for health education specialists. Int J Environ Res Public Health.

[34] Kimmons R, Veletsianos G (2018). Public internet data mining methods in instructional design, educational technology, and online learning research. TechTrends.

